# Effect of Environmental Temperature on the Content of Impurity Li_3_V_2_(PO_4_)_3_/C in LiVPO_4_F/C Cathode for Lithium-ion Batteries

**DOI:** 10.3389/fchem.2018.00283

**Published:** 2018-07-24

**Authors:** Taotao Zeng, Changling Fan, Zheng Wen, Qiyuan Li, Zeyan Zhou, Shaochang Han, Jinshui Liu

**Affiliations:** ^1^College of Materials Science and Engineering, Hunan University, Changsha, China; ^2^Hunan Province Key Laboratory for Advanced Carbon Materials and Application Technology, Hunan University, Changsha, China

**Keywords:** lithium-ion batteries, lithium vanadium fluorophosphates, environmental temperature, alveolate structure, electrochemical performance

## Abstract

Previous studies have shown that the impurity Li_3_V_2_(PO_4_)_3_ in LiVPO_4_F will adversely affect its electrochemical performance. In this work, we show that the crystalline composition of LiVPO_4_F/C is mainly influenced by the environmental temperature. The content of Li_3_V_2_(PO_4_)_3_ formed in LiVPO_4_F/C is 0, 11.84 and 18.75% at environmental temperatures of 10, 20, and 30°C, respectively. For the sample LVPF-30C, the SEM pattern shows a kind of alveolate microstructure and the result of selected area electron diffraction shows two sets of patterns. The LiVPO_4_F/C cathode without impurity phase Li_3_V_2_(PO_4_)_3_ was prepared at 10°C. The selected area electron diffraction result proves that the lattice pattern of LiVPO_4_F is a regular parallelogram. Electrochemical tests show that only one flat plateau around 4.2 V appears in the charge/discharge curve, and the reversible capacity is 140.4 mAh·g^−1^ at 0.1 C, and 116.3 mAh·g^−1^ at 5 C. From these analyses, it is reasonable to speculate that synthesizing LiVPO_4_F/C at a low environmental temperature is a practical strategy to obtain pure crystalline phase and good electrochemical performance.

## Introduction

The rechargeable lithium-ion battery has been widely studied because of its applications in electric vehicles, mobile phones, and energy storage devices (Huang et al., 2009; Konarov et al., 2017). LiFePO_4_ delivers superior thermal stability and excellent cyclic performance, but a low working potential decreases its energy density (Yamada et al., 2003; Kim et al., 2015; Eftekhari, 2017; Wu et al., 2017).

A novel cathode lithium vanadium fluorophosphate (LiVPO_4_F) material has been reported (Gover et al., 2006). The working potential (4.2 V) of LiVPO_4_F is much higher than that of LiFePO_4_ and LiCoO_2_ (Ma et al., 2013a; Hu et al., 2014; Wu et al., 2016). Moreover, the thermal stability of LiVPO_4_F is better than that of LiFePO_4_ and LiCoO_2_ (Wang et al., 2014; Xu et al., 2015). If the shortcoming of electronic conductivity is solved, LiVPO_4_F will be an outstanding cathode material (Reddy et al., 2010; Ma et al., 2013b; Satish et al., 2016). Some improvements have been adjusted to LiVPO_4_F cathode, such as cation doped, carbon coated and various synthesized routes (Wang et al., 2013a; Liu et al., 2016; Wu et al., 2018). Recently, adopting facile and controllable methods to prepare LiVPO_4_F is the key areas of research. LiVPO_4_F was reported by two-step carbothermal reduction in some references. However, this method suffers from high energy consumption and a large content of carbon, because the intermediate VPO_4_ is prepared separately at 700–800°C (Ma et al., 2014; Liu et al., 2015; Wang et al., 2016).

Thus, a novel one-step method in which the synthesis of VPO_4_ is omitted and carbon content is restricted to a very low level is of great research interest. Although the electrochemical performance of LiVPO_4_F prepared is improved, the plateaus belonging to impurity Li_3_V_2_(PO_4_)_3_ come into being (Liu et al., 2012; Wang et al., 2013b; Xiao et al., 2013). Therefore, the formation of Li_3_V_2_(PO_4_)_3_ is observed even though we use a synthesis method that employs a novel chemical reduction route. The content of Li_3_V_2_(PO_4_)_3_ should be carefully controlled because it may adversely affect the performance of the LiVPO_4_F cathode.

In this work, we discovered that the formation of impurity Li_3_V_2_(PO_4_)_3_ is directly related to the environmental temperature. The formation mechanism was investigated through further analysis of the structure and synthesis procedures.

## Experimental

### Materials synthesis

LiVPO_4_F/C was synthesized by using a novel chemical reduction method. The chemical reagent used was of analytical reagent grade. 0.03 mol H_2_C_2_O_4_ dissolved in deionized water was used as a chelating agent and reducing agent. 0.01 mol V_2_O_5_ was added slowly under vigorous magnetic stirring at 60°C. LiF and NH_4_H_2_PO_4_ at the molar ratio of 1:1 to vanadium were introduced in after 10 min. A PVDF carbon source of 1.4943 g was dispersed in 30 ml water in a solution of hexadecyl trimethyl ammonium bromide under ultrasonic assistance at 50°C. Subsequently, the PVDF suspension was added to the reaction system. Finally, the suspension was dried overnight in vacuum at 85°C. The precursor was presintered at 400°C for 5 h and sintered at 800°C for 4 h in a tubular furnace with flowing high-purity argon.

### Characterization

The crystal structure of the material was examined by X-ray diffraction (XRD, Rigaku D/MAX 2500). The morphology and elemental content were investigated with scanning electron microscopy (SEM, Navo NanoSEM230) and energy disperse spectroscopy (EDS). Nanoscale morphology and selected area electron diffraction (SAED) were performed by using high-resolution transmission electron microscopy (HRTEM, JEOL-3010).

### Electrochemical test

The electrochemical performance of LiVPO_4_F/C electrodes was evaluated using an Arbin BT2000 battery test system. The cathode film was fabricated by mixing LiVPO_4_F/C (80 wt.%), acetylene black (15 wt.%), and PVDF (5 wt.%) in the solvent N-methyl pyrrolidone, and the slurry was coated on an aluminum collector. The electrodes were dried in a vacuum oven at 120°C for 12 h and 2016 coin-type cells were assembled in a glove box (S1220/750). The electrolyte was 1.3 mol·L^−1^ LiPF_6_ in a mixing solvent of ethylene carbonate, dimethyl carbonate, and ethyl methyl carbonate (1:1:1). A lithium foil and a polypropylene separator (Celgard 2400) were used as counter electrode and separator, respectively.

## Results and discussion

The electrochemical performance of triclinic LiVPO_4_F/C is partially determined by the content of impurity Li_3_V_2_(PO_4_)_3_/C in it. Our study revealed that LiVPO_4_F prepared at a high environmental temperature delivers poor performance. To investigate the reason for this, we synthesized LiVPO_4_F/C at different environmental temperatures (30, 20, and 10°C), and named the respective samples as LVPF-30C, LVPF-20C, and LVPF-10C.

**Figure 1:**
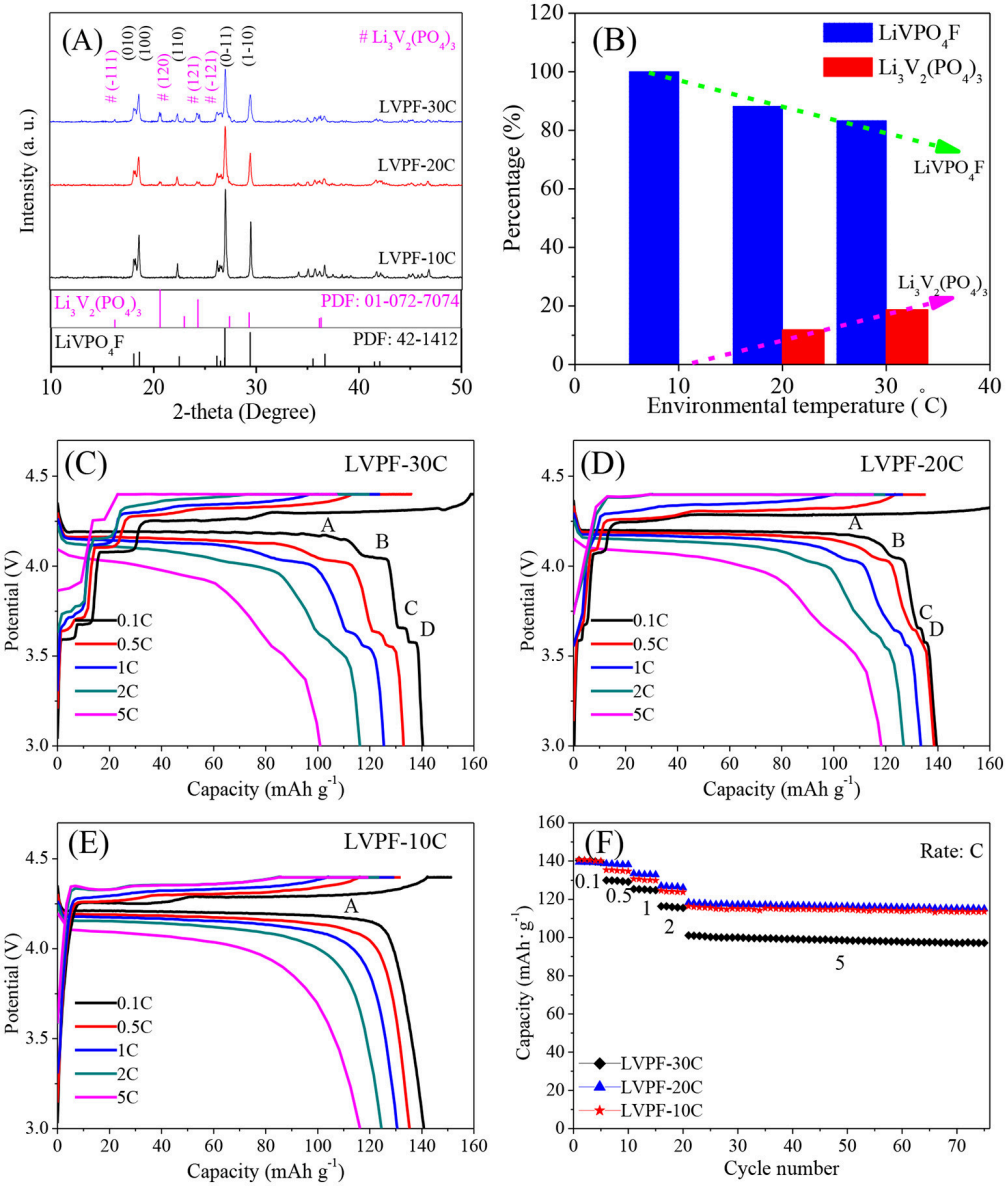
XRD patterns of LiVPO_4_F/C **(A)**, percentage of Li_3_V_2_(PO_4_)_3_ in LiVPO_4_F/C **(B)**, and charge/discharge curves of LiVPO_4_F/C **(C–F)**.

The XRD patterns of the samples are shown in Figure 1A. The main diffraction peaks correspond to a triclinic system with the space group of P-1, and can be indexed as the standard pattern of LiVPO_4_F (Barker et al., 2003; Huang et al., 2009). The absence of peaks corresponding to crystalline carbon proves that carbon is amorphous. No impurity peaks in LVPF-10C, which delivers the strongest peaks among the samples, was found. The refined cell parameters a, b, and c were 5.174, 5.308, and 7.509 Å, and the cell volume was 174.18 Å^3^. These results compare well with the classic results reported by Barker (Barker et al., 2005). However, the peaks at 20.69°, 23.53°, and 24.48° belonging to the impurity Li_3_V_2_(PO_4_)_3_ (symbol # in Figure 1A) occur in the curves of LVPF-30C and LVPF-20C (Zhu et al., 2008). The percentages of Li_3_V_2_(PO_4_)_3_ in LiVPO_4_F were estimated by refining the XRD patterns in Figure 1B. The content of Li_3_V_2_(PO_4_)_3_ increased gradually from 0% (10°C) to 11.84% (20°C) and 18.75% (30°C). Hence, our preliminary presumption is that low environmental temperature plays an important role in the preparation of pure LiVPO_4_F.

In Figure 1C, four flat plateaus (A, B, C, and D) appear in the discharge curves of LVPF-30C. The predominant plateau A around 4.2 V is attributed to LiVPO_4_F/C, and is in accordance with Barker's work (Barker et al., 2003), and B, C, and D are assigned to Li_3_V_2_(PO_4_)_3_/C. The specific capacities at 0.1 C and 5 C are 138.6 and 101.1 mAh·g^−1^. However, when temperature drops to 20°C (corresponding to LVPF-20C, Figure 1D), the plateaus of Li_3_V_2_(PO_4_)_3_/C are shorter than before, establishing the decreasing content of impurity. The specific capacity increases obviously, especially at 5 C (118.3 mAh·g^−1^). Further, only a plateau A at 4.2 V without other plateaus of impurity Li_3_V_2_(PO_4_)_3_/C is observed in LVPF-10C (Figure 1E). It is important to note that Li_3_V_2_(PO_4_)_3_/C disappear entirely. The specific capacities at 0.1 C 1 C and 5 C are 140.4 mAh·g^−1^, 130.6 mAh·g^−1^ and 116.3 mAh·g^−1^, which are very close to that of LVPF-20C in Figure 1F. LVPF-30C delivers the worst performance at a high current density. LVPF-20C with 11.84% impurity Li_3_V_2_(PO_4_)_3_/C possesses the optimum capacity at a high current density. The reason is that Li_3_V_2_(PO_4_)_3_/C is a fast ion conductor and allows a fast transfer of lithium ions in the cathode. Nevertheless, an excess of the impurity Li_3_V_2_(PO_4_)_3_/C in LiVPO_4_F/C adversely affects the rate and the cycling capability.

**Figure 2:**
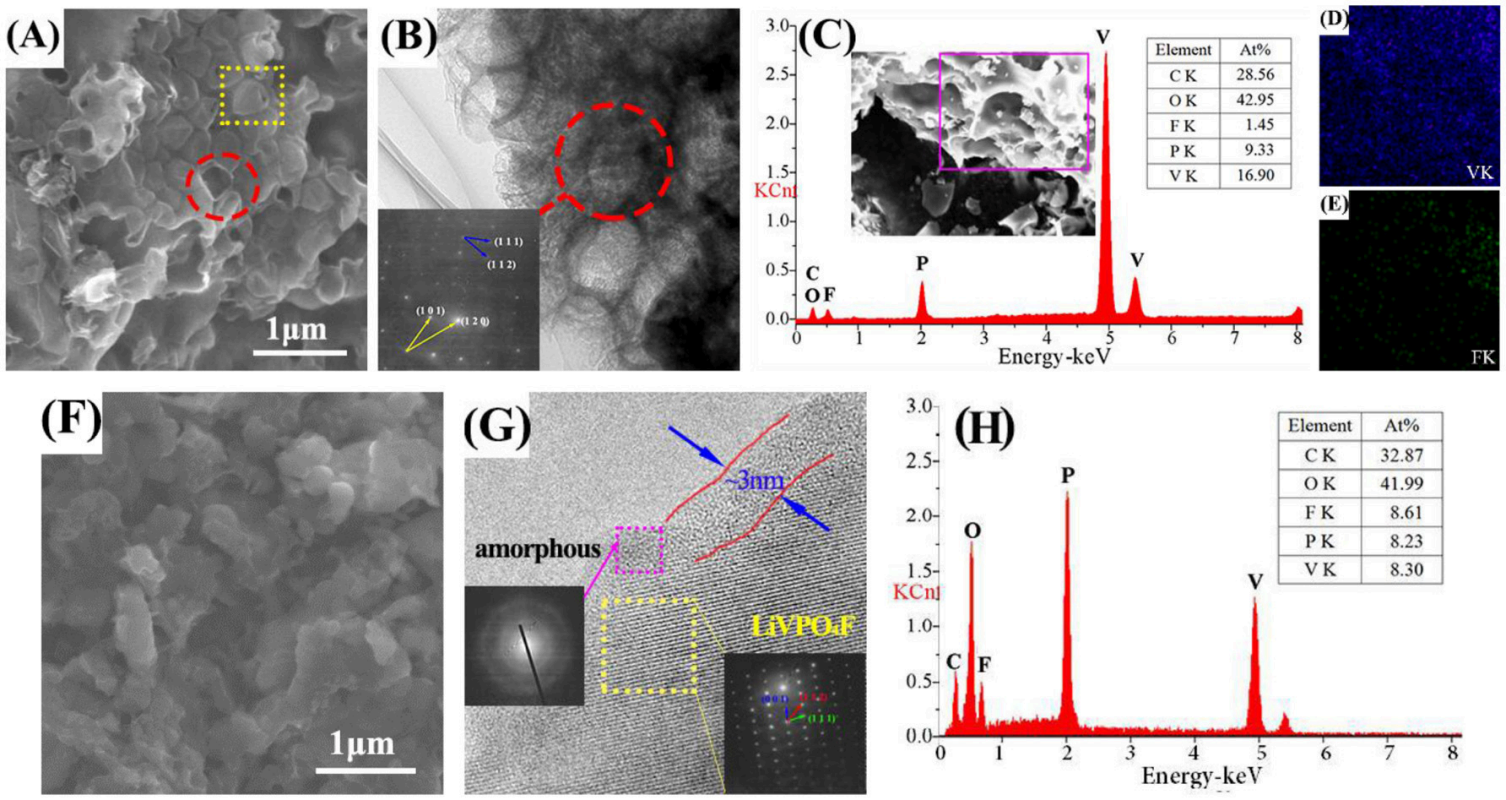
SEM, HRTEM, SAED, and EDS images of LVPF-30C **(A–E)** and LVPF-10C **(F–H)**.

In Figure 2A, the alveolate structure can be easily observed in LVPF-30C. The surface of most particles is broken. This structure is observed in the HRTEM image. The SAED pattern is made up of two sets of lattices with different characteristics (inset of Figure 2B). These parallelogram lattices are attributed to triclinic LiVPO_4_F (bottom) and monoclinic Li_3_V_2_(PO_4_)_3_ (top). In Figure 2C, the EDS image in the alveolate field proves the existence of Li_3_V_2_(PO_4_)_3_ distinctly because the content of fluorine is much lower than that of vanadium. Figures 2D,E show that vanadium is uniformly distributed on the surface of particles and a small quantity of fluorine is detected. This proves that impurity Li_3_V_2_(PO_4_)_3_ without fluorine is formed in the alveolate zone.

There is no alveolate structure on the flawless surface of LVPF-10C (Figure 2F) and the lattice fringes can be clearly observed (Figure 2G). The pattern of SAED in the square frame is a typical parallelogram, and is similar to the bottom lattice in Figure 2B. This pattern is attributed to the typical crystalline form of LiVPO_4_F with the triclinic system. Thus, LVPF-10C possesses a good crystalline morphology with a thin layer covering on the surface of the crystalline LiVPO_4_F. Its lattice pattern is a series of concentric circles, which is the characteristic of amorphous carbon (Song et al., 2008). The atomic contents of vanadium and fluorine are 8.61 and 8.30%, respectively, and match well with the atomic ratio of LiVPO_4_F in Figure 2H. Thus, we conclude that low temperature (10°C) helps to prepare pure phase LiVPO_4_F.

**Figure 3:**
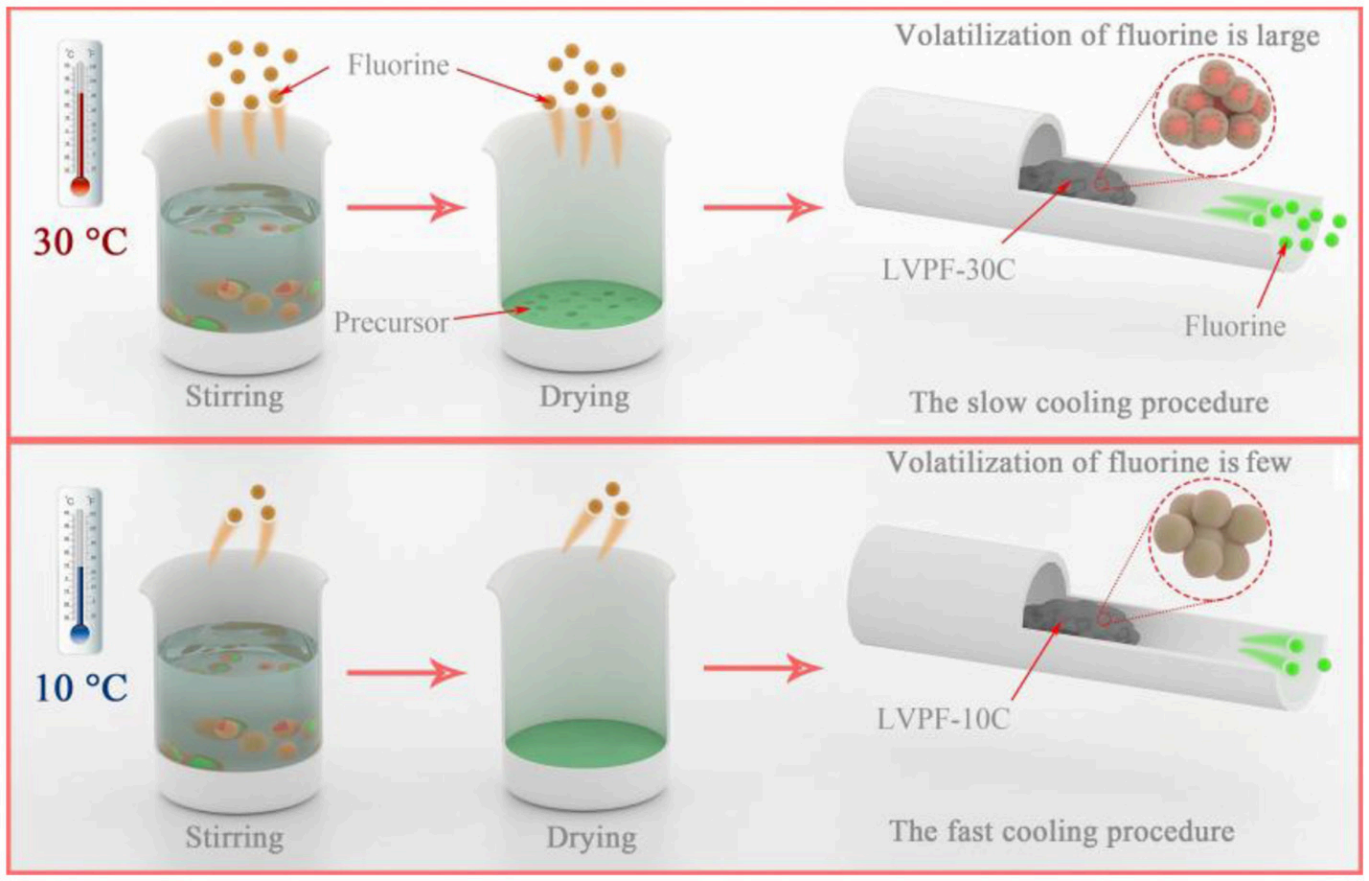
Schematic of the volatilization of fluorine at environmental temperatures of 30°C and 10°C.

The formation mechanism of the alveolate structure is investigated in Figure 3. On one hand, the excessive oxalic acid hydrolyzes in deionized water and produces hydrogen ions in aqueous solution. Ammonium dihydrogen phosphate generates ammonium ions in the hydrolysis reaction. A fluoride compound is formed when a hydrogen ion and an ammonium ion are combined with a fluoride ion released by LiF. Therefore, HF and NH_4_F are formed in the reaction (Zhou et al., 2009). It is well known that fluoride compounds are unstable and easily evaporate. From the viewpoint of reaction kinetics, the volatilization rate of fluoride will increase at least 6 to 8 times at 30°C compared to 10°C in reaction and drying. Therefore, the content of fluorine in the precursor at 30°C is evidently lower than that at 10°C. It can be inferred that the impurity Li_3_V_2_(PO_4_)_3_ is formed in this condition. On the other hand, the temperature of the tubular furnace drops slowly at 30°C. The cooling rate of LVPF-30C is lower than that of LVPF-10C. The longer cooling time of LVPF-30C accelerates the evaporation of fluoride, especially at 600–800°C. Therefore, this ensures that the fluorine content in LVPF-30C is much less than the value determined. The impurity Li_3_V_2_(PO_4_)_3_ is formed, which is in accordance with the above analysis of its structure and morphology.

Hence, the volatilization of fluoride should be inhibited in the preparation processes of LiVPO_4_F. Based on all of the evidence we have presented above, we legitimately conclude that a lower environmental temperature is more helpful to synthesize a LiVPO_4_F/C cathode with a low content of impurity and excellent electrochemical performance.

## Conclusions

A sample of LVPF-10C, which was prepared at an environmental temperature of 10°C, exhibited a regular parallelogram space pattern that is attributed to the pure triclinic form of LiVPO_4_F. High environmental temperature accelerates the volatilization of fluoride in the drying and sintering process and decreases the fluorine content. Then, a large quantity of Li_3_V_2_(PO_4_)_3_ reduces the plateaus in the discharge curves and deteriorates the rate of performance in LVPF-30C. Therefore, our work is devoted to give a direction to improve the synthetic process and advise what we need to do in the future.

## Author contributions

TZ wrote the paper and designed the main part of the experiment. CF was the main advisor. ZW and QL carried out material preparation and the electrochemical test. ZZ discussed and refined the paper. TZ, CF, ZW, QL, and ZZ proposed the research. CF, SH, and JL obtained the main financial support for the research and supervised all the experiments.

### Conflict of interest statement

The authors declare that the research was conducted in the absence of any commercial or financial relationships that could be construed as a potential conflict of interest.
